# Changes in Uric Acid Levels and Effects on Renal Function After Switching From Febuxostat to Dotinurad in Patients With Chronic Kidney Disease: A Retrospective Study

**DOI:** 10.7759/cureus.83332

**Published:** 2025-05-02

**Authors:** Akira Mima, Keishi Matsumoto, Tatsumasa Matsuki, Takaaki Morikawa, Sakura Kure, Ryosuke Akai, Yuta Saito, Shinji Lee

**Affiliations:** 1 Nephrology, Osaka Medical and Pharmaceutical University, Takatsuki, JPN

**Keywords:** chronic kidney disease (ckd), dotinurad, estimated glomerular filtration rate, febuxostat, uric acid

## Abstract

Dotinurad is a novel and selective uric acid (UA) reabsorption inhibitor. On the other hand, febuxostat, a xanthine oxidase inhibitor, reduces UA production, but its UA-lowering effect is limited. Therefore, this study will compare UA levels to baseline values after switching from febuxostat to dotinurad in hyperuricemic patients with chronic kidney disease (CKD), and will evaluate the effects on renal function and safety.

Eight eligible patients with CKD who had been treated with febuxostat (10-20 mg/day) for more than six months and then switched to dotinurad (0.5-4 mg/day) were studied retrospectively. Changes in UA levels, the percentage of patients achieving a UA level below 6.0 mg/dL, and changes in estimated glomerular filtration rate (eGFR) were analyzed.

Serum UA did not change significantly with the switch from febuxostat to dotinurad, but the rate of UA levels below 6.0 mg/dL was maintained or achieved in 63% of cases. The eGFR slope tended to slow with the change to dotinurad, but the difference was not statistically significant. Furthermore, no severe side effects were observed in any of the patients during the observation period.

In CKD patients with hyperuricemia, the change from febuxostat to dotinurad did not cause a significant change in UA levels. The eGFR slope tended to slow with the change to dotinurad, although the difference was not statistically significant.

## Introduction

Uric acid (UA) is a crucial endogenous metabolite in humans. It is generated in the liver as the final product of purine metabolism and is also taken in from food and other sources. Furthermore, UA is eliminated via the kidneys and intestines. Hyperuricemia is usually characterized by a UA level of 7.0 mg/dL or higher [1]. Excess UA in the blood can form crystals that accumulate in the joints and induce inflammation [2]. This state can cause gouty arthritis, which increases the risk of chronic kidney disease (CKD) initiation and progression [3]. Most Japanese patients with high UA levels show low UA excretion, which has been believed to be partially attributable to the progression of CKD and a decline in glomerular filtration rate (GFR), resulting in elevated serum UA levels due to reduced UA excretion [4]. However, a recent study revealed that Japanese people are, in fact, genetically predisposed to reduced UA excretion [5]. Despite this background, UA production inhibitors are mainly used for the treatment of hyperuricemia, while uricosuric agents are rarely used.

UA excretion transporters include urate anion transporter 1 (URAT1), located in the proximal tubule and responsible for UA reabsorption, as well as ATP-binding cassette subfamily G member 2 (ABCG2), organic anion transporter 1 (OAT1), and organic anion transporter 3 (OAT3), which are also involved in UA excretion [6,7]. Interestingly, these UA excretion transporters are involved in the renal excretion of both UA and uremic substances, such as indoxyl sulfate [8]. It has been reported that uremic substances may accumulate in various organs, including the kidney, which is a risk factor for CKD [9]. ABCG2 is expressed in the kidney and intestinal tract and is involved not only in UA excretion but also in uremic toxin excretion in CKD [10]. However, it has been reported that long-term use of febuxostat may decrease ABCG2 expression [11].

To the best of our knowledge, no studies have examined the effect of changing from febuxostat to dotinurad on UA levels and renal function in CKD patients with hyperuricemia. Therefore, this study was undertaken to elucidate these points.

## Materials and methods

Study design and collection of medical data

This study retrospectively reviewed and analyzed medical records. The patients received outpatient care or were hospitalized at the Department of Nephrology, Osaka Medical and Pharmaceutical University, Takatsuki, Japan. Of the 214 patients diagnosed with hyperuricemia, follow-up data were not available for two. In addition, there was one death and one case in which adverse effects unrelated to dotinurad administration were confirmed. Three patients were excluded because they were on dialysis.

**Figure 1:**
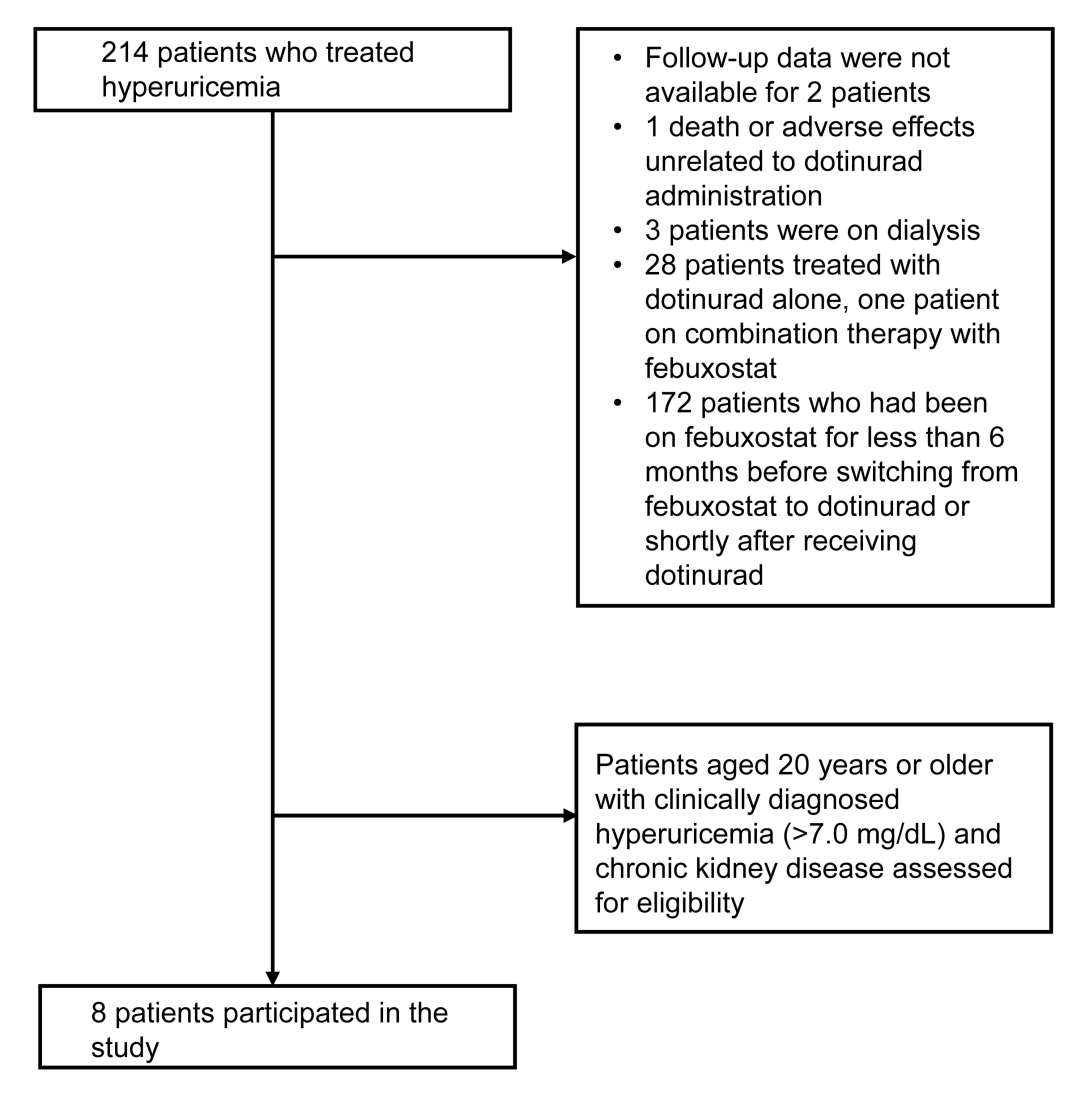
Flow diagram depicting the selection of eligible patients

Furthermore, 28 patients were treated with dotinurad alone, one patient received combination therapy with febuxostat, and 172 patients who had been on febuxostat for less than six months before switching to dotinurad or shortly after receiving dotinurad were excluded. Of the excluded patients, a total of eight aged 20 years or older were clinically diagnosed with CKD and hyperuricemia (>7.0 mg/dL), making them eligible participants (Figure 1). The procedures conducted with human participants in this study adhered to the ethical standards outlined by the National Research Committee and the 1964 Helsinki Declaration, along with its subsequent amendments or equivalent ethical norms. The Ethics Committee of Osaka Medical and Pharmaceutical University approved this study (approval number: 2022-144).

Retrospective data collection and analysis were performed using electronic medical records from Osaka Medical and Pharmaceutical University. Blood and urine biochemical analyses were conducted with a Labospect 008 autoanalyzer (Hitachi, Tokyo, Japan). Data on serum UA, serum creatinine, and patient characteristics - including medication, age, and sex - were extracted from the electronic medical records [12-14]. The estimated GFR (eGFR) for each patient was calculated using the four-variable MDRD (Modification of Diet in Renal Disease) formula, traceable to isotope dilution mass spectrometry (IDMS), along with the three-variable Japanese equation: $194 \times \text{serum creatinine} - 1.094 \times \text{age} - 0.287 \times 0.739$ (if female) [14-17].

Data analysis

Continuous variables are presented as mean ± standard deviation. The Wilcoxon test was applied to analyze variations in clinical data. All analyses were conducted with StatView (SAS Institute, Cary, CA, USA) and Excel software (Microsoft® Corp., Redmond, WA, USA), and statistical significance was set at p < 0.05.

## Results

Table 1 shows the baseline characteristics of the eight patients (four males and four females). The average age at onset was 75.1 ± 9.8 years. All the patients were treated with febuxostat (15 ± 5.3 mg/day) before switching to dotinurad (1.6 ± 1.1 mg/day). Concomitant medications were as follows: sodium-glucose cotransporter-2 (SGLT2) inhibitors (50%), ezetimibe (12.5%), statins (12.5%), dipeptidyl peptidase-4 inhibitors (12.5%), tirzepatide (12.5%), tolvaptan (25%), furosemide (12.5%), hypoxia-inducible factor-prolyl hydroxyl domain inhibitors (12.5%), angiotensin receptor blockers (12.5%), calcium channel blockers (37.5%), α-blockers (25%), and angiotensin receptor-neprilysin inhibitor (37.5%).

**Table 1:** Patient characteristics SGLT2, sodium-glucose cotransporter-2; DPP-4, dipeptidyl peptidase-4; HIF-PHIs, hypoxia-inducible factor-prolyl hydroxyl domain inhibitors; ARBs, angiotensin II receptor blockers; Ca-blockers, calcium channel blockers; ARNI, angiotensin receptor neprilysin inhibitor

Parameter	Value
Number of patients	8
Sex ratio (male:female)	4:04
Age (years, mean ± SD)	75.1 ± 9.8
Treatment dosage of febuxostat (mg/day, mean ± SD)	15 ± 5.3
Treatment dosage of febuxostat (mg/day, mean ± SD)	1.6 ± 1.1
Concomitant drugs	
SGLT2 inhibitors (n)	4
Ezetimibe (n)	1
Statins (n)	1
DPP-4 inhibitors (n)	1
Tirzepatide (n)	1
Tolvaptan (n)	2
Furosemide (n)	1
HIF-PHIs (n)	1
ARBs (n)	1
Ca-blockers (n)	3
α-blockers (n)	2
ARNI (n)	3

**Figure 2:**
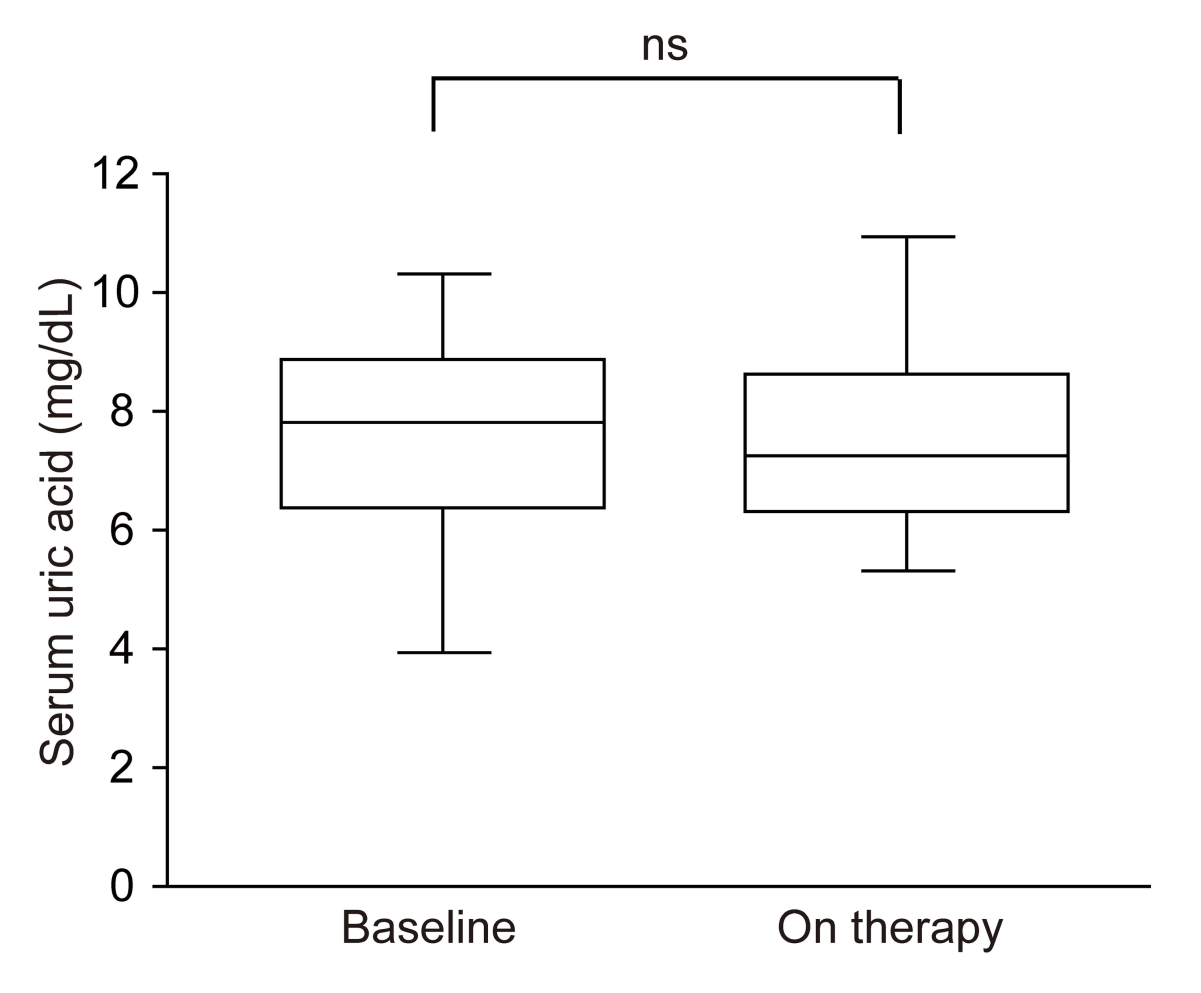
Box plots showing the levels of serum uric acid during febuxostat treatment and after switching to dotinurad treatment ns, not significant

There were no significant changes in UA levels with the change from treatment with febuxostat to dotinurad (before switching vs. after switching: 7.5 ± 2.4 and 7.6 ± 2.1 mg/dL, respectively; p = 0.673) (Figure 2 and Table 2).

**Table 2:** p-values for each parameter in this study The Wilcoxon test was applied to the analysis of clinical data, and statistical significance was set at p < 0.05. eGFR, estimated glomerular filtration rate

Variable	p-value
Uric acid	0.673
eGFR	0.674

**Figure 3:**
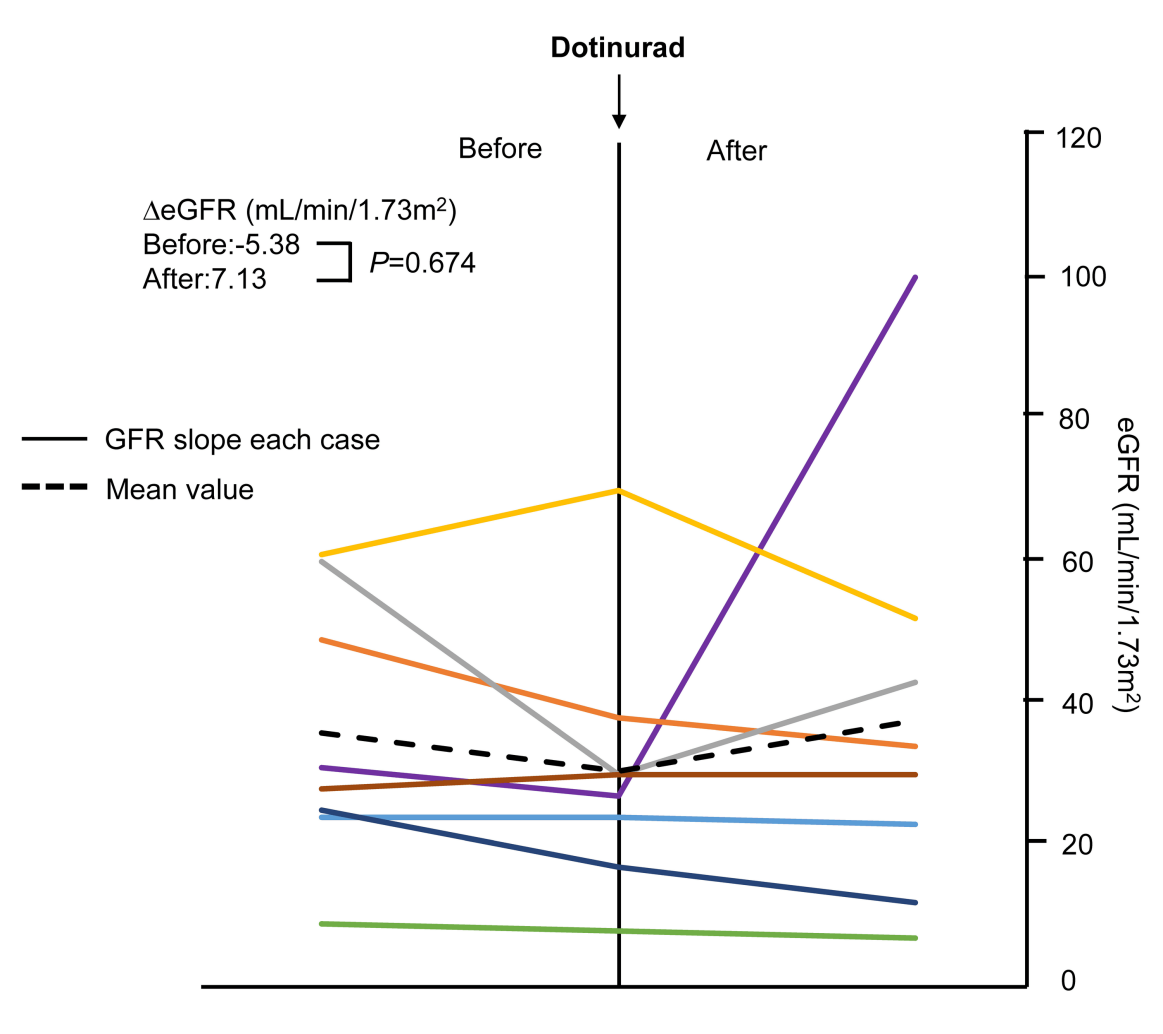
Slopes of eGFR rate before and after administration switching to dotinurad The same color in the left and right panels represents the same patients, and the black dashed line shows the mean value for all patients. GFR, glomerular filtration rate; eGFR, estimated glomerular filtration rate

The eGFR slope tended to decrease, albeit without statistical significance (before switching vs. after switching: -5.4 ± 12 and 7.1 ± 28 mL/min/1.73 m², respectively; p = 0.674) (Figure 3 and Table 2).

## Discussion

To the best of our knowledge, this study has shown the efficacy and safety of switching from febuxostat to dotinurad on serum UA in patients with CKD for the first time. Furthermore, the effect of changing from febuxostat to dotinurad on the eGFR slope was also examined.

Lesinurad, a URAT1 inhibitor, has been reported to have a high incidence of elevated serum creatinine and renal-related adverse events [18]. This is possibly due to the increased UA excretion and crystallization in the renal tubules. On the other hand, a long-term phase III study evaluating the safety of dotinurad showed no notable changes with respect to renal damage or renal parameters [19]. In addition, long-term treatment with dotinurad resulted in a significant improvement in renal function from baseline [20]. Furthermore, in our previous study with dotinurad in CKD patients with eGFR less than 25, dotinurad markedly reduced UA levels but had no effect on eGFR or urinary protein [21].

The effect of xanthine oxidase inhibitors on renal function is unclear from previous studies [22,23]. Against this background, our group investigated the effect of topiroxostat, a xanthine oxidase inhibitor, on renal function. However, our study also failed to demonstrate the renoprotective effect of xanthine oxidase inhibitors [24].

As CKD advances, the excretion of UA through ABCG2 in the intestine rises, which compensates for the excretion from the kidney; ABCG2 also plays a significant role in the elimination of uremic toxins like indoxyl sulfate [25]. In fact, previous studies indicated that ABCG2 knockout mice showed increased levels of uremic toxins, leading to the progression of CKD [8]. Disruption of the intestinal microbiota in CKD may worsen the condition by increasing uremic toxins [26]. In contrast, our recent research demonstrates that restoring the intestinal microbiota with prebiotics can slow the progression of CKD (Yasuzawa and Mima; unpublished data). Recently, the role of SGLT2 inhibitors in the treatment of CKD has become significant [27,28]. Notably, SGLT2 inhibitors enhance the transcriptional activity of the ABCG2 gene through the phosphorylation of cyclic adenosine monophosphate (cAMP) response element binding [29].

Several mechanisms have been proposed for the development of renal damage induced by UA, including the involvement of intraglomerular inflammation and nuclear factor kappa B (NF-κB) as important mechanisms for the development of CKD. The inactivation of these mechanisms by decreasing UA has been reported [30-32]. Furthermore, UA could decrease nitric oxide synthesis, leading to endothelial dysfunction. We have reported that activation of protein kinase C beta 2 (PKCβ2) in glomerular endothelial cells could inhibit insulin/insulin receptor substrate 1 (IRS1) signaling, which exacerbates CKD [33,34].

Moreover, the activation of protein kinase C in the glomeruli is associated with transforming growth factor-β signaling, which promotes the production of extracellular matrix (ECM) components like type IV collagen, leading to mesangial expansion [35,36]. Elevated levels of UA have been shown to heighten inflammation or oxidative stress, contributing to the accumulation of ECM [37]. As a result, tight control of UA levels is essential to prevent the progression of CKD.

However, this study may have limitations due to the number of patients and the fact that the retrospective analysis was performed on a small cohort of patients at a single institution; hence, further research is needed. Although this study demonstrates the efficacy and safety of switching from febuxostat to dotinurad in patients with CKD, further studies are needed.

## Conclusions

This retrospective study examined the impact of switching from febuxostat to dotinurad in patients with CKD. The results showed that the change from febuxostat to dotinurad may safely and effectively maintain a reduction in serum UA levels. Although there was no statistically significant difference, the eGFR slope appeared to be slower with the change to dotinurad compared to the eGFR slope with febuxostat. The renoprotective effect of switching from febuxostat to dotinurad was not clear in our study; therefore, further investigation is warranted.
